# Localization of photoperiod responsive circadian oscillators in the mouse suprachiasmatic nucleus

**DOI:** 10.1038/s41598-017-08186-5

**Published:** 2017-08-15

**Authors:** Tomoko Yoshikawa, Natsuko F. Inagaki, Seiji Takagi, Shigeru Kuroda, Miwako Yamasaki, Masahiko Watanabe, Sato Honma, Ken-ichi Honma

**Affiliations:** 1 Photonic Bioimaging Section, Hokkaido University Graduate School of Medicine, Sapporo, Japan; 2 Department of Chronomedicine, Hokkaido University Graduate School of Medicine, Sapporo, Japan; 3 Department of Anatomy and Neurobiology, Kindai University Faculty of Medicine, Osaka-sayama, Japan; 4 Department of Complex and Intelligent Systems, Future University Hakodate, Hakodate, Japan; 5 Research Institute for Electronic Science, Hokkaido University, Sapporo, Japan; 6 Department of Anatomy, Hokkaido University Graduate School of Medicine, Sapporo, Japan; 7 Research and Education Center for Brain Science, Hokkaido University, Sapporo, Japan

## Abstract

The circadian pacemaker in the suprachiasmatic nucleus (SCN) yields photoperiodic response to transfer seasonal information to physiology and behavior. To identify the precise location involved in photoperiodic response in the SCN, we analyzed circadian *Period1* and PERIOD2 rhythms in horizontally sectioned SCN of mice exposed to a long or short day. Statistical analyses of bioluminescence images with respective luciferase reporters on pixel level enabled us to identify the distinct localization of three oscillating regions; a large open-ring-shape region, the region at the posterior end and a sharply demarcated oval region at the center of the SCN. The first two regions are the respective sites for the so-called evening and morning oscillators, and the third region is possibly a site for mediating photic signals to the former oscillators. In these regions, there are two classes of oscillating cells in which *Per1* and *Per2* could play differential roles in photoperiodic responses.

## Introduction

The circadian pacemaker in mammals is located in the suprachiasmatic nucleus (SCN) and responds to light and darkness to entrain to the day-night alternation (LD). The SCN circadian pacemaker also responds to changes in day length (photoperiod) and transfers seasonal information to physiology and behavior^1^. The SCN consists of a number of oscillating cells which are coupled with each other to produce coherent SCN output rhythms^2^. The core molecular mechanism involves a transcriptional and translational feedback loop composed of clock genes, *Per1*, *Per2*, *Cry1*, *Cry2*, *Bmal1*, *Clock*, and their protein products^3^. They show robust circadian rhythms in their expression.

Photoperiodic changes in behavior, especially in the length of activity time in nocturnal rodents, were ascribed to the phase-relation between two circadian oscillators which differentially respond to light and regulate the onset and end of activity band of circadian behavior rhythms^4^. The two oscillators are called respectively the evening (E) and morning (M) oscillators. Previously, we demonstrated different responses of the circadian rhythms in clock gene *Per1* expression to a long (LD 18:6) and a short (LD 6:18) day in the SCN of mice carrying a bioluminescence reporter^5^. The findings supported the hypothesis of the E and M oscillators and further suggested the site of two oscillators; the E oscillator in the anterior and the M oscillator in the posterior SCN, respectively. We also found the third oscillator in the anterior SCN, but the role of this oscillation in the photoperiodic response was not elucidated. In our previous study, the SCN was sectioned in a coronal plane, which could interrupt the connection of these oscillators located in the anterior and posterior SCN, and thereby could manifest two different oscillations unless otherwise tightly coupled. If this is the case, the manifestation of two oscillations might be an artifact of slicing as demonstrated between the dorsal and ventral SCN^6^. This possibility should be examined by horizontal sectioning of the SCN, in which the neural connections between the anterior and posterior SCN would largely be kept intact. On the other hand, the connection of two oscillations might be kept in the horizontally sectioned SCN, and under these conditions the artifact issue would be ignored.

One of the difficulties to identify the precise sites of the E and M oscillators stems from a slight change in the shape of an SCN slice during culturing. Because of the technical issue, statistical analysis of circadian rhythms at distinct sites of the SCN has not been successfully performed. To overcome the difficulty, we adopted a geometrical transformation method^7, 8^ to normalize the shape of a cultured SCN slice, which enabled us to statistically compare bioluminescence images from different sites of the SCN on pixel level.

In the present study, the circadian rhythms in *Per1* and PER2 were measured *ex vivo* by using bioluminescence reporters from horizontally sectioned SCN of mice exposed to a long or short day. We measured *Per1* transcript rhythm which allows us to compare the present results with our previous study^5^, and PER2 protein rhythm to compare our result with those from other groups^9–11^. And the phase-relationship between the two rhythms was compared under different photoperiods. Here we demonstrate the two oscillating regions which correspond respectively to the site of the E, M oscillators and an oscillator which may transfer light signals to the former two oscillators.

## Results

### *Per1-luc* and PER2::LUC bioluminescence throughout the horizontally sectioned SCN slices

**Figure 1:**
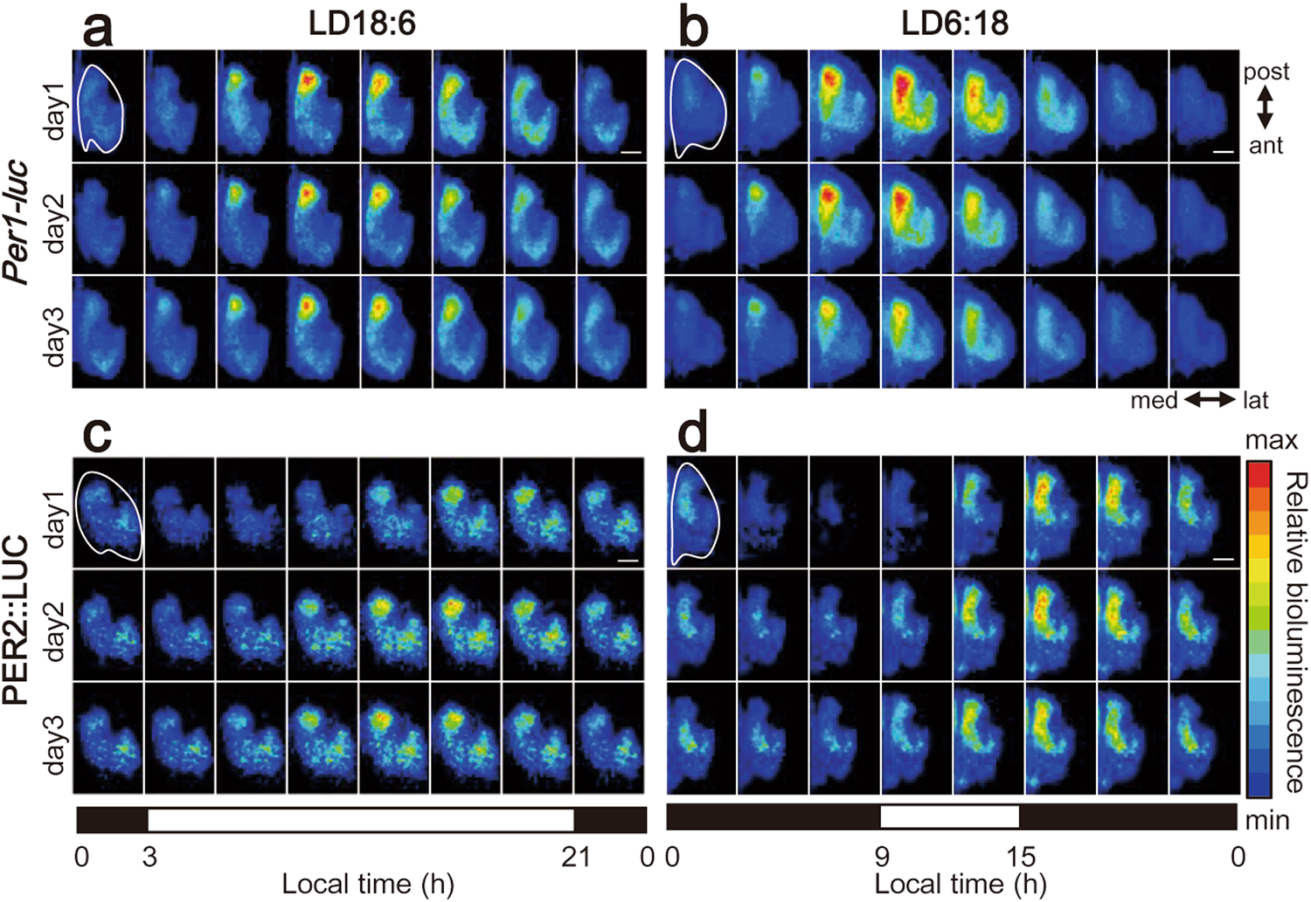
*Per-1uc* and PER2::LUC bioluminescence throughout the horizontally sectioned SCN slices. (**a**,**b**,**c**,**d**) Representative bioluminescence images of *Per1-luc* (**a,b**) and PER2::LUC (**c,d**) in the horizontally sectioned SCN slices of mice exposed to LD18:6 (left) or LD8:6 (right). The right SCN slices are illustrated with pseudocolor at every 3 h intervals on day 1, 2 and 3 in culture. A color bar in the right margin indicates relative strengths of bioluminescence. A white trace line in the top left image indicates the margin of the SCN. Scale bar, 50 μm. The black and white horizontal bars underneath each panel indicate respectively the dark and light phases on the day of tissue harvest.

To confirm the regional differences in the photoperiodic response detected by coronal sectioning of the SCN^5^, we re-examined those differences using horizontally sectioned SCN slices. Horizontal SCN slices of 100 μm thick were prepared from the adult *Per1-luc* ^5^ and *Per2* ^*Luc*^ ^12^ mice which had been exposed to a long (LD18:6) or short (LD6:18) day for 3–5 weeks. The slices were obtained at ca. 300 μm above the bottom of the brain, which expected to include the three targeted oscillators as suggested in the previous study^5^. *Per1-luc* and PER2::LUC bioluminescence from cultured SCN slices were analyzed on pixel level (pixel size: 3.7 × 3.7 μm). Bioluminescence of both *Per1-luc* and PER2::LUC was clearly detected as a seahorse shape with the head at the posterior end of the horizontal SCN slice, showing robust circadian rhythms at least for several days, especially at the head (Fig. 1, Supplementary movie).

**Figure 2:**
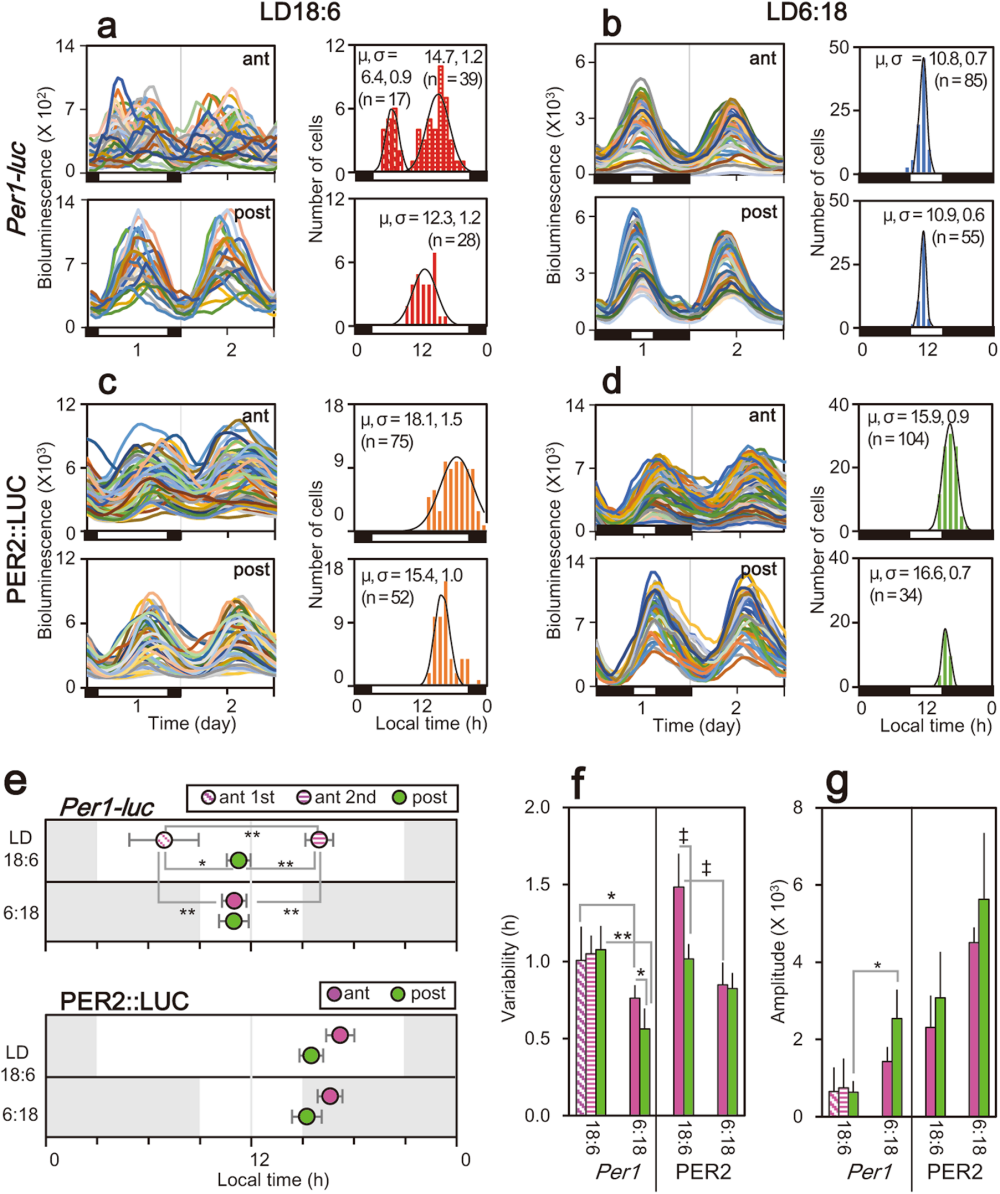
Circadian phase, variability of circadian peak and amplitude of the bioluminescence rhythms in cell-size regions of interest (ROI). (**a**,**b**,**c**,**d**) Forty-eight hour profiles of bioluminescence rhythms in cell-seize ROI and temporal distributions of circadian peaks on the first culture day for *Per1-luc* (**a**,**b**) and PER2::LUC (**c**,**d**) of the anterior (upper panel) and posterior (lower panel) SCN in each photoperiods. A single or double Gaussian curve fitted to a histogram of circadian phase is indicated by a black curve superimposed on a histogram. Median (μ) and variance (σ) of a Gaussian curve are inserted in each panel. (**e**) Mean medians (n = 5) in the anterior (pink circle) and posterior (green circle) SCN with SD. Two medians detected for the circadian *Per1-luc* rhythm in the anterior SCN from LD 18:6 are labelled as the 1st and 2nd peak. Shaded and white areas are the dark and light phases of LD on the day of tissue harvest. (**f**) Variability of circadian peak in terms of variance of Gaussian curve was evaluated in the regions of the SCN as well as in different photoperiods for *Per1-luc* (left) and PER2::LUC (right). Colors of vertical columns mean the clusters of circadian rhythms indicated in the panel e. (**g**) Mean amplitudes were similarly illustrated to the panel f of circadian rhythms obtained by CCD with a comparable sensitivity (*Per1-luc*: LD18:6, n = 4; LD6:18, n = 3. PER2::LUC: LD18:6, n = 3; LD6:18, n = 3). (**e–g**) **P < 0.01, *P < 0.05, one-way ANOVA with a post-hoc Tukey-Kramer test. ^‡^P < 0.01, two-way ANOVA with a post-hoc Tukey-Kramer test.

Circadian rhythms on the first culture day were analyzed in cell-size regions of interest (ROI) in the SCN slices (n = 20) (Fig. 2 and Supplementary Fig. 1). The circadian peak-phases were calculated by a geometric method reported previously^5^ and plotted against the local time in a histogram (Fig. 2a–d, right). With respect to the circadian *Per1-luc* rhythm, the distribution of peak phases in LD18:6 was bimodal in the anterior SCN, clustering at the beginning and the latter half of light phase (Fig. 2a, upper). The bimodality was confirmed by fitting to a double Gaussian equation to the distribution curve^13^ (Fig. 2a, right). By contrast, the peak-phase distribution in the posterior SCN in LD18:6 (Fig. 2a, lower) was unimodal. The same was true for both the anterior and posterior SCN in LD 6:18 (Fig. 2b). They were fitted to a single Gaussian equation. With respect to the circadian PER2::LUC rhythm, the peak-phase distribution was unimodal both in the anterior and posterior SCN, regardless of the photoperiod (Fig. 2c,d).

The mean medians (μ) of a fitted double Gaussian equation for *Per1-luc* in LD18:6 were located at 6.9 ± 1.0 h (mean ± SD, n = 5) and at 16.0 ± 1.1 h in the anterior SCN, whereas the mean median was detected at 11.3 ± 1.1 h in the posterior SCN (Fig. 2e, upper). The phases of three medians were significantly different (One-way ANOVA with a post-hoc Tukey-Kramer test, P = 2.07 × 10^−6^). In LD6:18, the mean median was located at similar phases in the anterior and posterior SCN (ant, 11.0 ± 0.8 h; post, 11.0 ± 0.6 h), while in LD6:18 the median phase in the anterior SCN was significantly different from the two anterior medians in LD18:6 and located just in between (one-way ANOVA with a post-hoc Tukey-Kramer test, P = 2.12 × 10^−6^) (Fig. 2e, upper). The median phase in the posterior SCN was not different between LD18:6 and LD 6:18. The mean median for PER2::LUC in LD18:6 was located at 17.2 ± 1.3 h in the anterior and at 15.5 ± 0.8 h in the posterior SCN (Fig. 2e, lower), while that in LD6:18 was located at 16.6 ± 1.1 h in the anterior and at 15.2 ± 1.0 h in the posterior SCN, respectively. Two-way ANOVA revealed significant differences between the anterior and posterior SCN in both rhythms, but did not between the photoperiods (region, P = 0.01; LD, P = 0.43; region × LD, P = 0.75).

We then compared the variability of a peak-phase distribution in terms of the mean variance (σ) of a fitted Gaussian equation. With respect to the circadian *Per1-luc* rhythms, the variability was significantly larger in LD18:6 than in LD6:18, regardless of the SCN region (one-way ANOVA with post-hoc Tukey-Kramer test, ant, P = 0.04; post, P = 9.68 × 10^−4^) (Fig. 2f, left). In LD6:18, the variability was significantly larger in the anterior SCN than in the posterior (one-way ANOVA with post-hoc Tukey-Kramer-test, P = 0.04). With respect to the circadian PER2::LUC rhythm, the variability in LD18:6 was significantly larger in the anterior SCN than in the posterior, and as for the anterior SCN it was significantly larger in LD18:6 than in LD 6:18 (two-way ANOVA; region, P = 4.33 × 10^−3^; LD, P = 3.76 × 10^−5^; region × LD, P = 8.26 × 10^−3^, with a post-hoc Tukey-Kramer test) (Fig. 2f, right).

The mean amplitude of circadian rhythm was obtained by averaging the individual means of circadian rhythm in ROI examined in each SCN slice (n = 5). The amplitude was expressed as a difference between the maximum and minimum values of circadian rhythm. With respect to the *Per1-luc* rhythms (Fig. 2g, left), the mean amplitude was not significantly different within the two SCN regions in either LD18:6 or LD6:18. On the other hand, the amplitude in the posterior SCN was significantly higher in LD6:18 than in LD18:6 (one-way ANOVA, P = 0.01). With respect to the circadian PER2::LUC rhythm (Fig. 2g, right), the mean amplitude was significantly larger in LD6:18 than in LD18:6 without significant difference between the regions (Two-way ANOVA; region, P = 0.28; LD, P = 0.02; region × LD, P = 0.84).

### Regional specificities of circadian *Per1-luc* rhythms

**Figure 3:**
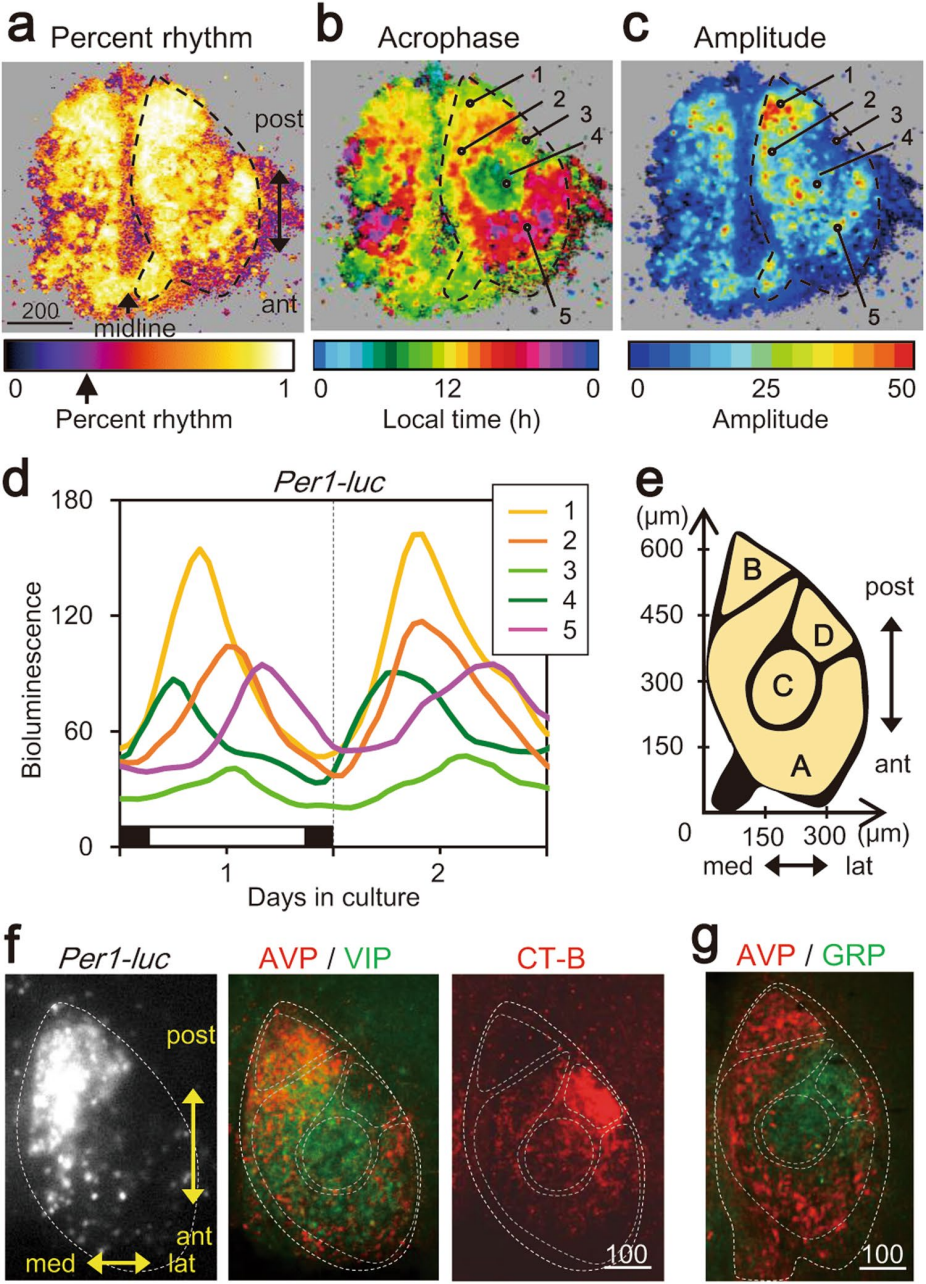
Regional specificities of circadian *Per1-luc* rhythms. (**a**,**b**,**c**) Pixel-level images of the percent rhythm (**a**), circadian peak (**b**) and rhythm amplitude (**c**) are illustrated throughout an SCN slice on the culture day 1. Color bars indicate the level of significance with an arrow indicating a significance level of P = 0.01 (**a**), local time (**b**) and height of amplitude (**c**). The pixels with lower bioluminescence than the background level were shown in grey. The pixels in which a significant circadian rhythm was not detected by percent rhythm test (arrow in a) are shown in black in b and c. A dashed trace line in each panel indicates the margin of the right SCN determined by immunohistochemical analysis. Scale bar, 200 μm. (**d**) Bioluminescence rhythms in five ROIs (circle with a 3-pixel diameter) shown in the panel b and c. (**e**) Based on panels a, b and c, the four SCN regions of distinct circadian characteristics are schematically drawn in the right SCN. (**f**) Image of bioluminescence (left), immunofluorochmistry of AVP and VIP double staining (middle), and of the retinohypothalamic projections visualized by Alexa 594 conjugated cholera toxin subunit B (CT-B, right) of an SCN slice in *Per1-luc* mouse exposed to LD18:6. The CT-B and bioluminescence images were taken at 13:00 and 18:00, respectively on the day of slice preparation. The slice was fixed at 16:00 on the 6th day in culture. Margin of the SCN and the regions A-D are shown by broken trace lines. V, third ventricle; OC, optic chiasm. (**g**) Image of immunofluorochmistry of AVP and GRP. The SCN slice was fixed at 12:00 in LD18:6.

In order to systematically evaluate the photoperiodic responses in different regions of the SCN, we measured bioluminescence on pixel level (pixel size: 3.7 × 3.7 μm). Temporal changes of *Per1-luc* bioluminescence were analyzed with a cosine curve fitting method using data obtained at one hour intervals. Significance of circadian rhythm was tested by a percent rhythm method^14^ (Fig. 3a). The acrophase (peak phase) and amplitude of circadian rhythm were obtained from the best fitted cosine curve of significant fitting (P < 0.01) (Fig. 3b,c).

In LD18:6, almost all pixels indicated significant circadian rhythms, showing the highest fitting in the posterior SCN (Fig. 3a). With respect to the circadian peak, sharply demarcated three regions were detected in the acrophase map (Fig. 3b). The acrophase in the posterior region of the SCN (head of seahorse) was located at around the middle of the light phase, that in the open-ring-shaped region (body and tail) was at around 18:00 h, and that in the oval centrum surrounded by the open-ring was at around 8:00 h (Fig. 3b). The amplitude map revealed the highest amplitude in the posterior SCN and lowest in the centrum to the lateral outlet (Fig. 3c). ROI-based circadian *Per1-luc* rhythms in the typical regions mentioned above confirmed the general feature of circadian rhythm in each region (Fig. 3d).

Based on these characteristics of the acrophase and amplitude maps, we divided the SCN into four regions and named them alphabetically (Fig. 3e): the region A, an open-ring-shape region blank at the posterolateral margin of the SCN, covering the anterior to posterior part of the SCN. In this region, the circadian rhythms were most phase-lagged and the rhythm amplitude was intermediate among the four regions; the region B, a triangle region at the posterior end where the phases of circadian rhythms were intermediate and the amplitude was highest among the four regions; the region C, the oval centrum surrounded by the open-ring, where the circadian rhythms were most phase-ahead and the amplitude was low; the region D, the outlet of the open-ring, where the circadian peak phases were similar to those of the region C and the amplitude was the lowest.

These regions were superimposed with the areas of immunohistochemically stained neuropeptides. The region A and B were found to cover the arginine-vasopressin (AVP) positive area, while the region C and a part of region A covered the vasoactive intestinal peptide (VIP) positive area (Fig. 3f). A part of the region C and the region D covered the gastrin releasing peptide (GRP) positive area. The region D also covered the cholera toxin subunit B (CT-B) positive area where the retinohypothalamic tract projected (Fig. 3f,g).

### Statistical evaluation of acrophase and amplitude of the four regions in the horizontal SCN slice

**Figure 4:**
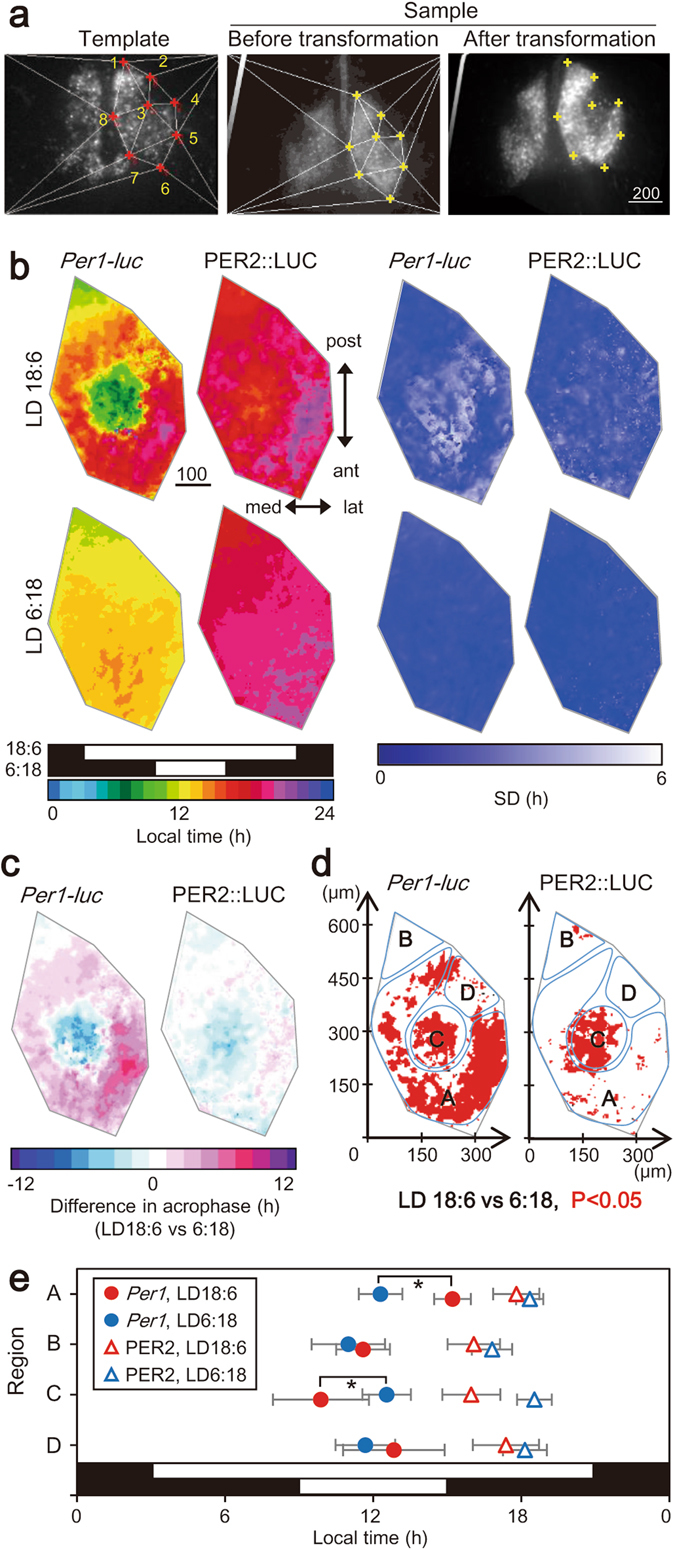
Statistical evaluation of acrophase of *Per1-luc* and PER2::LUC rhythms on pixel level. (**a**) Geometric transformation of an SCN bioluminescence image for statistical analyses on pixel level; a template SCN (left), a sample SCN before (middle) and after (right) the transformation. Red and yellow crosses with white lines (Delaunay triangles) in the panels indicate eight reference points in the template (1-8) and a sample SCN. (**b**) The mean acrophase map composed of five standardized images of individual SCN slices (Supplementary Fig. 2) are illustrated for *Per1-luc* (far left) and PER2::LUC (left) rhythms. Respective SD map is demonstrated in the right half. Horizontal black and white bars indicate light and dark phases of the photoperiod (upper, LD18:6; lower, LD6:18) with local time. SD values are indicated by pseudocolor bar. (**c**) Mean phase difference between circadian rhythms in LD18:6 and LD 6:18 is illustrated in an SCN map for *Per1-luc* (left) and PER2::LUC (right). A pseudo-color bar indicates the phase-difference in hour for phase-advance with a negative sign and for phase-delay with a positive. (**d**) Statistical significance of a phase difference was demonstrated in an SCN map for each pixel. Red marks indicate the pixels with significant phase difference between LD18:6 and LD6:18 (t-test, P < 0.05). (**e**) Mean acrophases with SD in the four SCN regions (Fig. 3e) for the circadian *Per1-luc* (circle) and PER2::LUC (triangle) rhythms in LD18:6 (red) and LD6:18 (blue). Numbers of pixel included in the regions are 5938 (A), 1063 (B), 2436 (C) and 1064 (D). *P < 0.01, LD18:6 vs. LD6:18, two-way ANOVA with a post-hoc Tukey-Kramer test.

In order to confirm the above mentioned regional feature is also the case for other SCN slices and to perform statistical evaluation of circadian rhythms in these regions, we standardized bioluminescence images of the all cultured SCN slices examined (n = 35 including all slices in this study). For this purpose, we adopted a geometrical transformation using Delaunay triangulation^8^ and affine mapping^7^. The transformation was done without any assumption of structural specificities of the regions. Eight reference points were adopted in the margin of bioluminescence area of the template SCN slice selected (Fig. 4a). The rest of SCN slices were transformed according to the template SCN. Thus, all the SCN slices examined had common coordinates, which made statistical analyses of circadian rhythms possible on pixel level. The averaged image was demonstrated in Fig. 4b and each of the transformed SCN image was illustrated in Supplementary Fig. 2b,c (n = 20). The change in the size of these SCN by this transformation was 119 ± 24% on average.

We next examined the mean phase relationships, acrophases and amplitudes of circadian rhythms in different regions of the standardized SCN. The circadian *Per1-luc* rhythms in the regions B and C were significantly phase-ahead than that in the region A in LD18:6, whereas the regional difference was not detected in LD6:18 (Fig. 4b–d, Supplementary Fig. 3). By contrast, only a slight regional difference was observed in the circadian PER2::LUC rhythm, but the difference due to photoperiods was not clear. The phase-difference between LD18:6 and LD6:18 (Fig. 4c) was significant in the substantial number of pixels of the region A and C for the circadian *Per1-luc* rhythm, and in the most of the region C for the PER2::LUC rhythms (t-test, P < 0.05) (Fig. 4d). The phase-difference was essentially absent in the region B and D for the circadian *Per1-luc* rhythm, and the regions A, B and D for the circadian PER2::LUC rhythm.

The mean acrophases of circadian *Per1-luc* rhythm obtained in the four regions were significantly different in LD18:6 (one-way ANOVA, P = 1.50 × 10^−3^) but not in LD6:18 (P = 0.27) (Fig. 4e). When the acrophase in LD18:6 was compared with that in LD6:18, it was significantly phase-delayed in the region A by 2.9 h and significantly phase-advanced in the region C by 2.6 h (two-way ANOVA post-hoc Tukey-Kramer test, region, P = 2.46 × 10^−3^; LD, P = 0.31; region × LD, P = 2.93 × 10^−3^). The acrophase of the circadian PER2::LUC rhythm in LD18:6 was not different among the four regions (one-way ANOVA, P = 0.08) but was significantly phase-delayed in LD6:18 (two-way ANOVA, region, P = 0.01; LD, P = 1.89 × 10^−3^; region × LD, P = 0.14) (Fig. 4e).

**Figure 5:**
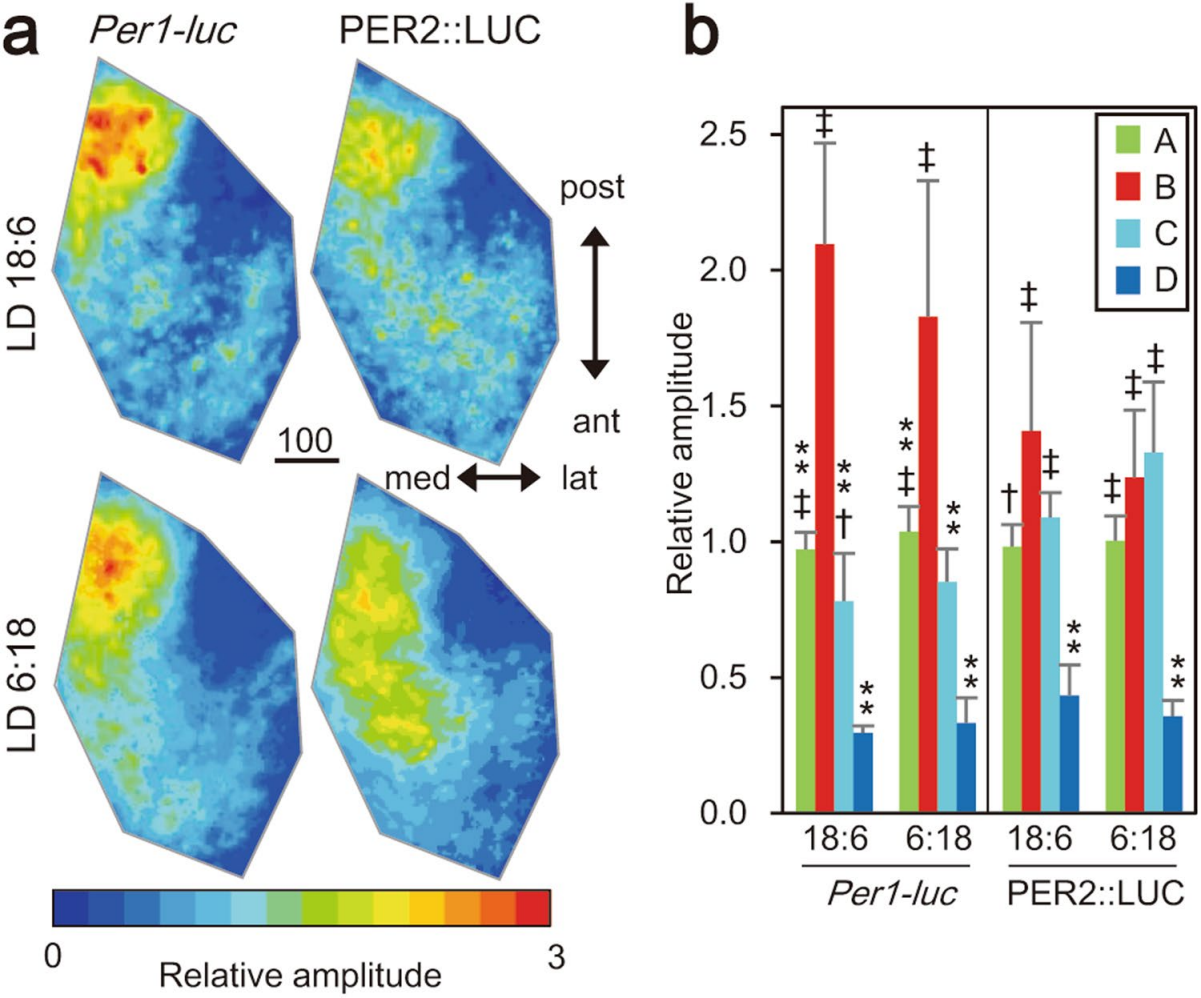
Regional difference in the amplitude of circadian *Per1-luc* and PER2::LUC. (**a**) Mean amplitude (n = 5) maps for the circadian *Per1-luc* (left) and PER2::LUC (right) rhythm in LD18:6 (upper) and LD6:18 (lower). Color bars indicate relative amplitudes expressed in a ratio to the mean of each SCN slice. Scale bar, 100 μm. (**b**) Mean relative amplitudes with SD in the four SCN regions for the circadian *Per1-luc* and PER2::LUC rhythms. **P < 0.01, vs. region B, ^‡^P < 0.01, ^†^P < 0.05 vs. region D, one-way ANOVA with a post-hoc Tukey-Kramer test.

The amplitude of circadian rhythm was also region specific. Relative amplitude to the mean of each SCN was calculated on pixel level (Fig. 5a). The means of relative amplitude in the four regions were shown in Fig. 5b. With respect to the circadian *Per1-luc* rhythm, there was a significant difference in the four regions in both LD18:6 and LD6:18, showing the highest mean amplitude in the region B and the lowest in the region D (one-way ANOVA, LD18:6, P = 1.53 × 10^−8^; LD6:18, P = 7.06 × 10^−6^). With respect to the circadian PER2::LUC rhythm, the regional difference in the amplitude was statistically significant in both LD18:6 and LD6:18, showing a lower amplitude in the region D than in other three regions in which the relative amplitude was not different from each other (one-way ANOVA; LD18:6, P = 9.75 × 10^−5^; LD6:18, P = 6.76 × 10^−6^; Fig. 5b).

### Blunted regional difference of acrophase in constant darkness

**Figure 6:**
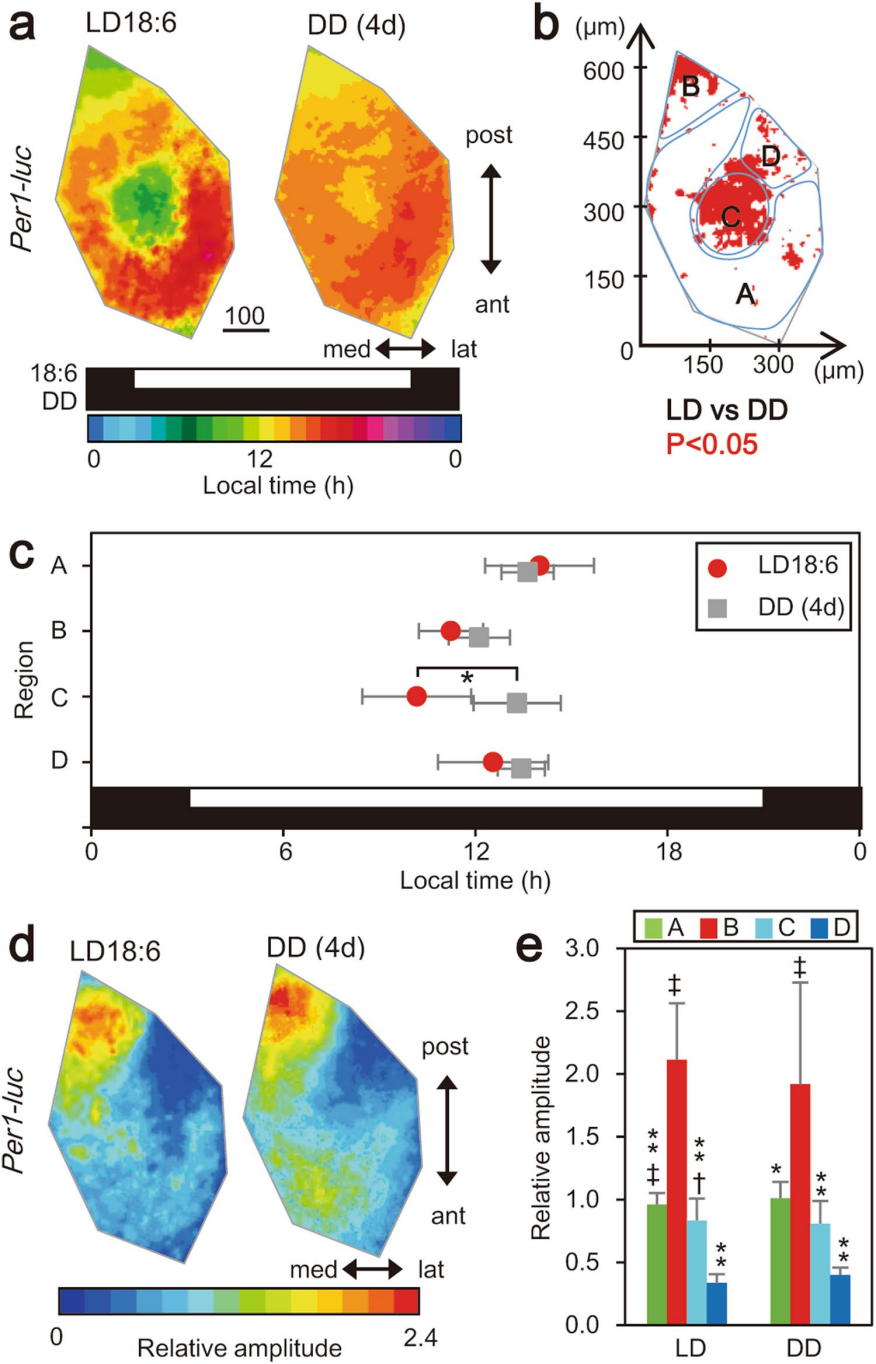
A blunted regional difference in acrophase of *Per1-luc* rhythm by exposure to constant darkness. (**a**) Mean acrophase maps for the circadian *Per1-luc* rhythm of the mice exposed to LD18:6 (left, n = 8) and of the mice exposed to DD for four days (DD (4d)) after LD18:6 (right, n = 7). Scale bar, 100 μm. Horizontal black and white bars indicate light and dark phases of photoperiod; the upper LD 18:6 and the lower DD. A color bar indicates local time. (**b**) Statistical significance of phase differences between LD18:6 and DD (4d) is demonstrated in an SCN map. Red marks indicate the pixel**s** with significant phase difference (t-test, P < 0.05). (**c**) Mean acrophases with SD in the four SCN regions (Fig. 3e) in LD18:6 (red circle) and DD (grey square). *P < 0.01, vs LD18:6, two-way ANOVA with a post-hoc Tukey-Kramer test. (**d**) Mean amplitude maps in LD18:6 and DD (4d). Color bars indicate relative amplitudes. See also the legend of Fig. 5. Scale bar, 100 μm. (**e**) Mean relative amplitudes with SD in the four SCN regions. **P < 0.01, *P < 0.05, vs. region B, ^‡^P < 0.01, ^†^P < 0.05 vs. region D, one-way ANOVA with a post-hoc Tukey-Kramer test.

To understand the intrinsic nature of regional difference in circadian phase, the phase-relation was examined without an influence of LD cycle. *Per1-luc* mice kept in LD18:6 were released into constant darkness (DD) for four days and the horizontally sectioned SCN were cultured for several days. Circadian parameters were compared between LD and DD. The regional difference in the acrophase observed in LD (one-way ANOVA, P = 5.67 × 10^−4^) disappeared in DD (one-way ANOVA, P = 0.06) (Fig. 6a–c). The regional difference in the relative amplitude of circadian *Per1-luc* rhythm persisted in DD with the highest amplitude in the region B and the lowest in the region D (one-way ANOVA; LD 18:6, P = 1.13 × 10^−12^; DD, P = 1. 76 × 10^−5^). (Fig. 6d,e).

### Distribution of *Per1* and *Per2* mRNA in the SCN

**Figure 7:**
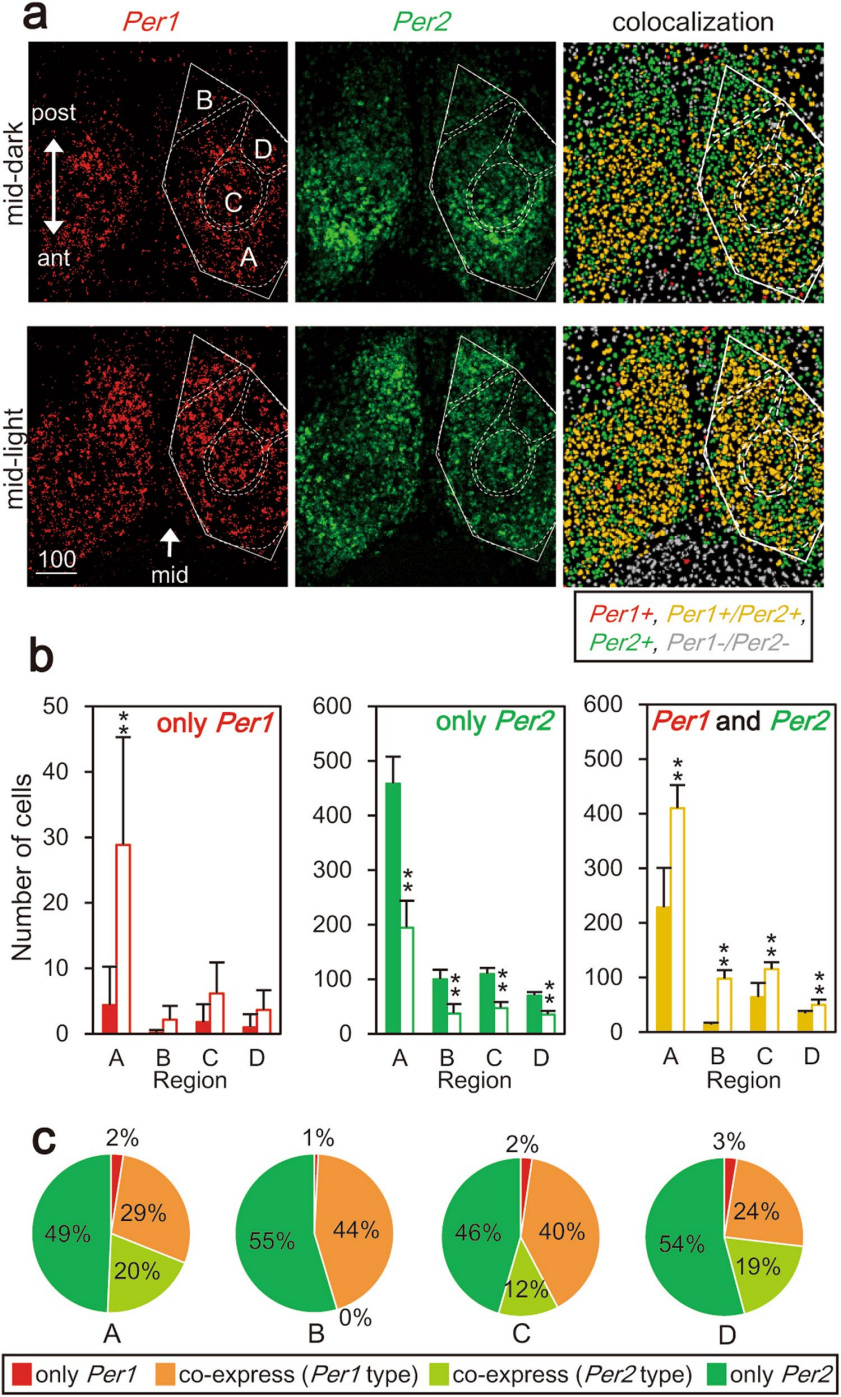
Distributions of *Per1* and *Per2* mRNA in the horizontal SCN slice. (**a**) Representative images of *Per1* (left, red) and *Per2* (middle, green) mRNA positive signals at the mid-dark (0:00 h in local time) and mid-light (12:00 h in local time) by double labeled *in situ* hybridization. Co-expression is shown in the right where double-positive and -negative cells are shown in yellow and grey, respectively. White solid and dashed trace lines are the margin of the SCN and of each SCN region (Fig. 3e), respectively. (**b**) Numbers of only *Per1*, only *Per2* and *Per1/Per2* expressing cells at the mid-dark (n = 5, filled bar) and mid-light (n = 6, open bar) are demonstrated in reach SCN region (mean and SD). The numbers of only *Per1* and only *Per2* expressing cells were obtained from the total numbers of *Per1*, *Per2* and *Per1/Per2* expressing cells. **P < 0.01, *P < 0.05, two-way ANOVA with a post-hoc Tukey-Kramer test. (**c**) Pie charts showing the percentages of different types of cells in the SCN slice. The cell types are classified based on the expression of circadian rhythm (see text); *Per1* only expressing cells (red), *Per1* type cells in co-expressing cells (orange), *Per2* type cells in co-expressing cells (light green) and *Per2* only expressing cells (green). Approximately 30% of the total cells showed the *Per1* type circadian rhythm in the region A and D, whereas more than 40% of total cells in the region B and C showed the *Per1* type circadian rhythm.

In order to understand the origin of differential photoperiodic responses between circadian *Per1-luc* and PER2::LUC rhythms, we examined the gene expressions on single cell level by double-labeling *in situ* hybridization (Fig. 7a). There detected three types of cells that were labeled differentially; the cells expressing only *Per1*, those expressing only *Per2* and the cells expressing both *Per1* and *Per2*. The only *Per1* expressing cells are rare and approximately 20 times less than the only *Per2* expressing cells (Fig. 7b). Approximately a half of SCN cells examined co-expressed *Per1* and *Per2*. The number of the only *Per1* expressing cells was predominant at the mid-day and the day/night ratio was 5.86 calculated for the entire SCN region (for the particular regions: A, 7.25; B, 11.00; C, 3.00; D, 4.00), whereas the number of the only *Per2* expressing cells was abundant at the mid-night and the day/night ratio was 0.43 for the entire SCN region (for the particular regions: A, 0.43; B, 0.37; C, 0.43; D, 0.50). The day/night ratio of the co-expressing cells was different from either of them, and was hard to be explained by a simple assumption that *Per1* and *Per2* are expressed rhythmically in the same single cell. These findings rather indicate that two different types of cells were mixed in the population of the co-expressing cells; one is the cells which showed the day/night pattern of the only *Per1* expressing cells without rhythmic changes in *Per2* expression (*Per1* type cell), and the other is the cells which show that of the only *Per2* expressing cells without rhythmic changes in *Per1* expression (*Per2* type cell). The numbers of *Per1* type and *Per2* type cells were calculated in each SCN region and the ratios to the total cell number of a respective region were illustrated in a pie chart together with the ratios of the only *Per1* and only *Per2* expressing cells (Fig. 7c). According to the chart, the ratio was different among regions. The circadian *Per1* rhythm was observed in approximately 31% of the total cells in the region A, 45% in the region B, 42% in the region C and 27% in the region D, respectively. Interestingly, almost all the co-expressing cells in the region B were the *Per1* type.

## Discussion

The present study revealed the sites of circadian oscillations which are involved in the photoperiodic response in the mouse SCN. The combination of pixel level analysis with a geometrical transformation method enabled us an objective and comprehensive analysis of time-lapse imaging data.

### Differential photoperiodic responses of circadian *Per1* and PER2 rhythms in the horizontal plane of the SCN slice

Previously, we demonstrated two clusters of cellular circadian rhythms of *Per1-luc* in the anterior SCN slice in the mice exposed to LD 18:6, but a single cluster in the mice to LD 6:18. On the other hand, in the posterior SCN, only one cluster of circadian rhythms was identified regardless of photoperiods. Consistent with these findings, the present study demonstrated two clusters of circadian rhythms in *Per1* expression in the anterior part of the horizontally sliced SCN in LD18:6 (Fig. 2a,b). It could be a matter of debate whether the change in the circadian system of the cultured SCN slice is an artifact of slicing or not, because slicing of the SCN could interrupt the neural network, resulting in disconnection of the regional oscillators. However, this possibility is less likely, because exactly the same results were obtained regardless of whether the SCN was sliced on the frontal plane^5^ or horizontal (Fig. 2a,b).

Surprisingly, the cellular circadian rhythms in PER2 showed a single cluster in the anterior part of the SCN regardless of whether they were exposed to LD18:6 or LD6:18 (Fig. 2c,d). The variability of a PER2 cell cluster in terms of the σ of Gaussian distribution was significantly larger in the anterior SCN in LD18:6 than those in LD6:18 (Fig. 2f). Recently, a similar photoperiodic variability of peak times of circadian PER2::LUC rhythm was reported^11^. These findings suggest the possibility that two clusters of cellular circadian rhythms are also involved in PER2 expression similar to *Per1* expression. Importantly, the phase position of circadian PER2 rhythms in reference to the light phase (or local time) was not changed by changes in photoperiod. As a result, the phase-angle difference from the circadian *Per1* rhythm was substantially changed in the anterior SCN under a long day (Fig. 2e). These findings indicate that the circadian rhythms in *Per1* expression and in PER2 are regulated by different oscillators.

### Four distinct regions in the horizontal SCN show characteristic responses to photoperiods

Geometrical transformation of the SCN slice by Delaunay triangulation^8^ and affine mapping^7^ revealed four distinct regions which are characterized by the circadian *Per1* rhythms of different parameters (Fig. 3e) and differential responses to photoperiods (Fig. 4b–e). Bioluminescence images of the horizontal SCN slice was characterized by the seahorse shape structure which was divided into two different regions; a major part of open-ring structure (region A) and a triangle region at the posterior end adherently to the ring (region B) (Fig. 3e). The circadian *Per1* rhythms in the region A had an intermediate amplitude and phase-shifted in response to the changes of photoperiod, corresponding to the phase-lagged circadian rhythms in the anterior SCN in LD18:6 (Fig. 2a,b). The circadian rhythms in the region B were highlighted by the highest amplitude and stable phases insensitive to the change of photoperiod, corresponding to the circadian rhythms in the posterior SCN (Fig. 2a,b). The region C is characterized by the circadian rhythms of intermediate amplitude and phase-shifting in response to the changes of photoperiod, corresponding to the phase-leading rhythms in the anterior SCN in LD18:6 (Fig. 2a). The region D is characterized by the circadian rhythms of the lowest amplitude and a lack of photoperiodic response (Figs 2–5). These regions could be reconstructed in serial sections of coronal SCN slices, since a rough picture of different regions were observable in the coronal slice of anterior SCN^5^.

Taken our previous and present results together, the circadian rhythms in the regions A and B (Fig. 4) could be regarded as the sites of the E oscillator driving the activity onset and of the M oscillator driving the activity offset, respectively. Two different circadian peaks were detected previously in neuronal activity of the horizontally sliced SCN in the hamster exposed to a long day^15^, supporting the above hypothesis. On the other hand, only one peak was detected in the SCN neuronal activity of mice which were exposed to either of LD12:12 or LD14:10^16^. The discrepancy could be due to a moderate long photoperiod (LD14:10) used in their study as compared with a longer photoperiod (LD18:6)^5^. The phase-angle difference between the two peaks could depend on the length of photoperiod^14^. The role of circadian rhythms in the region C were not characterized in the previous study^5^. A phase-relation of this oscillator to the light-on was almost the same in both photoperiods examined. In addition, the regional dependency of circadian phase was lost in DD without a significant change in the amplitude (Fig. 6). These findings suggest that the oscillator in the region C mediates the light signal to the E and M oscillators.

### Distribution of *Per1* and *Per2* expressing cells and differential responses to photoperiods

Interestingly, the circadian rhythms in PER2 did not respond to photoperiodic changes in day length except for the region C (Fig. 4c,d), partially consistent with the previous studies^9, 17^. Since the phase of circadian *Per1* rhythm was changed in the regions A and C, the phase-angle difference between the circadian *Per1* and PER2 rhythms in the region A, but not in the regions C, was significantly different in the two photoperiods (Fig. 4c,e). The finding indicates that the two circadian rhythms in this region are regulated by different oscillations. The notion is consistent with the previous report of different free-running periods of locomotor activity rhythm in *Per1* or *Per2* single knock-out mice^18^.

The majority of *Per1* expressing cells also express *Per2*, while there is a large cell population in which *Per2* are expressed without significant expression of *Per1* (Fig. 7), partially consistent with the previous reports of regional differences in the expressions of *Per1* and *Per2*, and their protein products^19, 20^. The present finding raises the question whether the photoperiodic changes in the phase-relationship between the two circadian rhythms occur within the same single SCN cells or not. We do not have any evidence for the hypothesis that the same single cells show different circadian oscillations for *Per1* and *Per2* expression. On the other hand, approximately one third of the *Per1* and *Per2* co-expressing cells showed the day/night expression pattern of *Per1* type in the region A (Fig. 7c). *Per2* expression was assumed not rhythmic in these cells. This assumption was based on an absence of the counterpart in the circadian PER2::LUC rhythm of the cell cluster that showed the circadian *Per1-luc* rhythms at the early light phase in LD18:6 (Figs 2a and 4b). Similar *Per1* type cell populations could be assumed in other regions (Fig. 7c). These finding suggests two classes of SCN cells in which *Per1* or *Per2* is exclusively involved in the circadian oscillation.

### Roles of neuropeptides in photoperiodic response

Each oscillating region is characterized by immunohistochemically identified neuropeptides. The region A included the AVP and VIP positive neurons, whereas the region B contained mostly the AVP positive neurons (Fig. 3f,g). On the other hand, the region C included the VIP and GRP positive neurons. **T**he region D contains the GRP neurons and was heavily labeled by CT-B, indicating the terminal of the retinohypothalamic tract. (Fig. 3f,g). VIP and AVP play important roles in integrating the cellular circadian rhythms in the SCN^21, 22^. A lack of VIP signaling in the SCN is known to disturb the circadian behavior rhythms to a various extent^13, 23^. VIP in the SCN seems to mediate the light signal from the retinohypothalamic tract to the AVP containing neurons^24, 25^ and its production was strongly affected by light^26^. On the other hand, the AVP containing neurons in the SCN are also important for the circadian behavior rhythms, especially for the coupling of the activity onset and offset^27^. In addition, AVP could be regarded as a mediator of the SCN neural network involved in build-up of cell clusters^28^. Thus, AVP and VIP are likely involved in photoperiodic changes in the phase-difference between the E and M oscillators.

In conclusion, there are at least four clusters of cellular circadian rhythms which are topologically distinct in the horizontal plane of the SCN and differentially respond to photoperiodic changes. The cell clusters located in the region A and B are corresponding to the E and M oscillators, respectively. The cell cluster in the region C possibly mediates the light signals from the retinohypothalamic tract to the E and M oscillators. The cell cluster in the region D is a site for receiving the light information from the retina. In these regions, there are two classes of oscillating cells which respond differently to photoperiod and in which *Per1* and *Per2* are differentially involved.

## Materials and Methods

Animal experiment was conducted in accordance with the Guidelines for the Care and Use of Laboratory Animals of Hokkaido University. The present experiments were approved by the Animal Research Committee of Hokkaido University (permission number 13-0069). Additional materials and methods are described in supplemental information.

## Electronic supplementary material

Supporting Information

Per1-luc bioluminescence throughout a horizontal SCN slice of the mouse exposed to LD18:6.

Per1-luc bioluminescence throughout a horizontal SCN slice of the mouse exposed to LD6:18.

PER2::LUC bioluminescence throughout a horizontal SCN slice of the mouse exposed to LD18:6.

PER2::LUC bioluminescence throughout a horizontal SCN slice of the mouse exposed to LD6:18.
