# Supplementary material for: Localization of photoperiod responsive circadian oscillators in the mouse suprachiasmatic nucleus
Source: Sci Rep. 2017 Aug 15;7:8210. doi: 10.1038/s41598-017-08186-5 (PMC5557852; doi:10.1038/s41598-017-08186-5)
Supplement: Supplementary file 1 — Supporting Information [file 41598_2017_8186_MOESM1_ESM.pdf]

Localization of photoperiod responsive circadian oscillators in the mouse suprachiasmatic nucleus

Tomoko Yoshikawa[1, 2, 3]*, Natsuko F. Inagaki[2], Seiji Takagi[4], Shigeru Kuroda[5], Miwako Yamasaki[6], Masahiko Watanabe[6], Sato Honma[2,7]*, Ken-ichi Honma[2]*
[1]Photonic Bioimaging Section, Research Center for Cooperative Project, [2]Department of Chronomedicine, Hokkaido University Graduate School of Medicine, [3]Department of Anatomy and Neurobiology, Kindai University Faculty of Medicine, [4]Department of Complex and Intelligent Systems, Future University Hakodate, [5]Research Institute for Electronic Science, Hokkaido University, [6]Department of Anatomy, Hokkaido University Graduate School of Medicine, [7]Research and Education Center for Brain Science, Hokkaido University

***Correspondence to:**
tomokoyn@med.kindai.ac.jp
sathonma@med.hokudai.ac.jp
kenhonma@med.hokudai.ac.jp

Supporting Information

Materials and Methods

Animals. Transgenic mice carrying a *Period1-luciferase* reporter (*Per1-luc* mice)[1] and knock-in mice carrying a PER2::LUC reporter (*Per2* ^{Luc} mice)[2] were bred and raised in our animal facility under 12:12 light dark cycles (LD 12:12) with lights on at 06:00 h and off at 18:00 h. The light intensity during the light phase was about 100 lux. Room temperature was 22 ± 2 °C, and humidity was 60 ± 10 %. Chow (Oriental Yeast Co., Tokyo, Japan) and tap water were available at all times. Weaning was done at the postnatal day 21 and only male mice were used for the experiments. At age 2-5 months old, mice were transferred to individual cages in light-tight boxes where light intensity was approximately 300 lux in the light phase.

In the experiment of bioluminescence imaging in two photoperiods, 20 mice were used (*Per1-luc* mice, n = 10, *Per2* ^{Luc} mice, n = 10). They were randomly assigned to the long or short day group (each n = 5). The number of animals was determined by a principle of the minimum number for the parametric evaluation of significance level, taking the general variance of circadian parameters into account. The photoperiod was changed to either LD 18:6 (long day, light-on at 3:00 and light-off at 21:00) or LD 6:18 (short day, light-on at 9:00 and light-off at 15:00) as described previously[1]. The mice were

exposed to either of the photoperiods for 3-5 weeks before the brain sampling for SCN slice culture. In the experiment of exposure to constant darkness (DD), *Per1-luc* mice (n = 15) were kept in LD 18:6 for 3 weeks, and randomly assigned to the long day or constant darkness (DD) group. The *Per1-luc* mice in the DD (n = 7) were subjected to SCN slice culture on the fourth day in DD. The mice kept in LD 18:6 (n = 8) were used as the control. In the experiments of immunohistochemical analyses, *Per1-luc* mice (n = 4) were used for staining AVP, VIP and GRP. To visualize the retinal projection to the SCN, *Per1-luc* mice (n = 5) were used for an anterograde tracer injection. The mice were kept in LD18:6 for 3 weeks before subjected to the experiments. In the experiment of fluorescence *in situ* hybridization (FISH), C57BL/6J mice (n = 12) were purchased from a local breeder and kept in our animal quarters for exposing to LD18:6 for 3 weeks before hybridization.

SCN slice culture and bioluminescence recording. Mice were decapitated and enucleated after cervical dislocation without anesthesia between 11:00 and 15:00 h. For mice in DD the above procedures were performed under infrared light (< 1 lux). The brain was removed and chilled in ice cold Hanks' balanced salt solution, which was followed by serial slicing of the brain tissue on a horizontal plane in 100 μm-thick with a microslicer (Dosaka EM, Kyoto, Japan). The brain slice was obtained from ca. 300

μm above the bottom of the optic chiasm, which contains the anterior tip to posterior end of the bilateral SCN. The brain slice was cultured on a membrane (Millicell-CM membrane, Millipore) with 1.3 ml of DMEM containing 0.2 mM D-luciferin K and 5 % culture supplements as previously reported[3]. Bioluminescence images were obtained using one of the following CCD cameras: ImagEM (Hamamatsu Photonics, Hamamatsu, Japan) cooled down to -80 °C, iXon3 (Andor, Belfast, UK) down to -80 °C and ORCA II (Hamamatsu Photonics) down to -60 °C. Bioluminescence was recorded every hour for at least 3 consecutive days. At the end of bioluminescence recording the brain slices were fixed with 4% paraformaldehyde in 0.1 M phosphate buffer (PFA) and subjected to immunohistochemical analysis as described below.

Data analyses of bioluminescence images. The day of slice preparation for culture was designated as day 0 in culture. Bioluminescence obtain from 0:00 h on day 1 in culture was subjected to the analyses of circadian rhythm parameters, unless otherwise mentioned. The local time was used for plotting the circadian rhythms.

For the ROI-level analyses, the peak phase of circadian rhythms were determined as described previously[1]. The amplitude of circadian rhythm was defined as a difference between the maximum and minimum level of the bioluminescence rhythm. The

horizontal plane of an SCN slice was divided into the anterior and posterior portions which contain approximately 60 and 40 % of the SCN, respectively. The temporal distribution of the peak phases in each ROI was examined in one hour bins and was fitted to a single or double Gaussian curve to obtain the median (μ) and variance (σ) of distribution[4] . The median and variance were calculated for each SCN slice of mice exposed to either LD18:6 or LD6:18. The mean median and variance were obtained for the circadian *Per1-luc* and PER2::LUC rhythms under different photoperiods (n = 5).

For pixel level analyses (pixel size, 3.7 × 3.7 μm) a time series of bioluminescence of each pixel was fitted to a cosine curve, as described previously[5]. Briefly, the background level of bioluminescence was subtracted from the original data. The background level was defined as the mean bioluminescence intensity of 9 pixels at the darkest corner of each image plus 5 SD of the mean. An acrophase and an amplitude (a double value of mathematical amplitude) of a best fitted cosine curve were used as the circadian peak phase and rhythm amplitude, respectively. Goodness of fitting was statistically evaluated by percent rhythm[6] at a significance level of $P < 0.01$. The circadian peak phase and amplitude were illustrated in pseudocolor.

For statistical comparison of the circadian rhythm parameters on pixel-level, the shape of SCN slice was normalized for each slice to obtain the mean bioluminescence

image. The shape of SCN image in each slice was geometrically transformed into the shape of template SCN slice which was selected beforehand. Eight reference points were placed at the margin of the bioluminescence (Fig. 4a). Those in the margins locate at: 1) the most posterior point, 2) the point where luminescence suddenly decrease at the postero-lateral corner, 3) the most medial point of the lateral dark area, 4) the point suddenly reappearing at the lateral border, 5) the most lateral point, 6) the most anterior point, 7) the antero-medial corner, 8) the most medial point. The points were mutually associated with the template points. Thus, the images were divided into 18 triangular regions by lines connecting these points (Delaunay triangulation)[7]. Each triangle was transformed into the corresponding triangle of the template by an affine mapping[8], designated as T. The pixel value at the position of each pixel position $(\mathrm{i,j})$ in the transformed image was obtained from the value at the corresponding pixel $(\mathrm{x,y})$ in the original image; the coordinate $(\mathrm{x,y})$ was calculated using the inverse affine mapping, designated as T^{-1}: i.e. $(\mathrm{x,y}) = T^{-1}(\mathrm{i,j})$. A pixel value at a point $(\mathrm{x,y})$ in the transformed image was calculated from the values of four adjacent points using the bilinear interpolation method. Afterwards, the cosine fitting analysis was applied to pixel values at a particular point of transformed image. To standardize bioluminescence intensity, the mean amplitude was calculated for all pixels in the SCN area, and used as

the reference to adjust the intensity.

Immunohistochemistry. The brain slices (n = 20) were fixed with 4% PFA after the bioluminescence recording, and immunohistochemically labeled with a cocktail of antibodies against AVP (AVP-NP, PS41[9]) and VIP (Peptide Institute, 14110) as described previously[5]. Other SCN slices (n = 4) were labeled with a cocktail of antibodies against AVP and GRP (Yanaihara Institute Inc., Y160). Immunohistochamical signals were examined by fluorescent microscopy (BZ9000; Keyence, Osaka, Japan).

To know the structural relationship between the region D and the terminals of the retinohypothalamic tract, an anterograde tracer of cholera toxin subunit B (CT-B) labeled with Alexa 594 (Molecular probes, C-22842) was injected into the bilateral vitreous bodies of *Per1-luc* mice via the sclera under ether anesthesia (n = 5). The mice were returned to their home cage for 48 h[10], and horizontal SCN slices were prepared as described above. The slices were placed in glass bottom culture dish and subjected to fluoromicroscopic observation of CT-B. Then the slices were transferred to culture dish and subjected to the bioluminescence imaging for more than 3 days. Finally, the slices were fixed with PFA for immunohistochemical analysis.

Double labeled fluorescent in situ hybridization. C57BL/6J mice (n = 12) were exposed to LD18:6 for three weeks. The brains were sampled at the mid-dark (0:00 h) or mid-light (12:00 h), frozen in crashed dry ice, and stored at -80 °C until further treatment. Frozen brain was sectioned serially (20 μm) by a cryostat (CM1900; Leica Microsystems), mounted on silane-coated glass slides and fixed as reported previously[11]. The sections were double-labeled by FISH with fluorescein-, and digoxigenin (DIG)-labeled cRNA probes[11] for *Per1* (390–1643; GenBank accession number AB030818) and *Per2* (1428-2953; NM011066) mRNAs, respectively. FISH was performed as described elsewhere[11]. Finally, sections were stained with NeuroTrace 640/660 Nissl stain (Invitrogen). One brain slice was missed in the course of preparation.

Photographs were taken with a confocal laser-scanning microscope (FV1200, Olympus, Tokyo, Japan). For the computer-based quantitative analysis of co-expression of *Per1* and *Per2*, we used the multi wavelength cell scoring plugin of the MetaMorph Software (Molecular Devices). One representative slice of which shape correspond to bioluminescence image was selected from each SCN. The numbers of *Per1* and *Per2* positive cells were counted in the regions A-D separately for bilateral SCN. The averages of six (mid-day) or five (mid-night) slices were calculated, and the day/night ratios for

each and the entire SCN region were obtained from the average values.

Analysis of circadian rhythms in *Per1* and *Per2* co-expressing cells. Using the day/night ratios of the number of only *Per1* expressing and only *Per2* expressing cells, the numbers of *Per1* type and *Per2* type cells in the co-expressing cell population were obtained by the following simultaneous linear equations:

$$\text{Tc-night} = N1 + N2 \times 1/R2$$

$$\text{Tc-day} = N1 \times R1 + N2$$

Tc-night: total number of co-expressing cells at midnight, Tc-day: total number of co-expressing cells at midday. N1: number of *Per1* type cell, N2: number of *Per2* type cell, R1; day/night ratio of only *Per1* expressing cells, R2; day/night ratio of only *Per2* expressing cells.

Statistical analyses. Statistical analyses were performed using Excel Tokei 2012 (SSRI, Japan). A one-way ANOVA with a post-hoc Tukey-Kramer test was used to evaluate differences in the same condition. A two-way ANOVA with a post-hoc Tukey-Kramer test was used to evaluate difference among different conditions. Bartlett's test was performed prior to ANOVA to confirm the variance among groups were equal. Two-sided

Student's t-test was used to evaluate difference between two groups in the pixel level analysis.

Reference

1. Inagaki, N., Honma, S., Ono, D., Tanahashi, Y. & Honma, K. Separate oscillating cell groups in mouse suprachiasmatic nucleus couple photoperiodically to the onset and end of daily activity. *Proc. Natl. Acad. Sci. U. S. A.* **104**, 7664-7669 (2007).
2. Yoo, S.-H., *et al.* PERIOD2::LUCIFERASE real-time reporting of circadian dynamics reveals persistent circadian oscillations in mouse peripheral tissues. *Proc. Natl. Acad. Sci. U. S. A.* **101**, 5339-5346 (2004).
3. Yoshikawa, T., *et al.* Daily exposure to cold phase-shifts the circadian clock of neonatal rats in vivo. *Eur. J. Neurosci.* **37**, 491-497 (2013).
4. Ono, D., Honma, S. & Honma, K.-i. Differential roles of AVP and VIP signaling in the postnatal changes of neural networks for coherent circadian rhythms in the SCN. *Science Advances* **2**, e1600960 (2016).
5. Yoshikawa, T., *et al.* Spatiotemporal profiles of arginine vasopressin transcription in cultured suprachiasmatic nucleus. *Eur. J. Neurosci.* **42**, 2678-2689 (2015).
6. Nelson, W., Tong, Y.L., Lee, J.K. & Halberg, F. Methods for cosinor-rhythmometry. *Chronobiologia.* **6**, 305-323. (1979).
7. O'Rourke, J. Voronoi Diagrams. in *Computational Geometry in C* 181-226 (Cambridge University Press, 1998).
8. Burger, W. & Burge, M.J. Geometric Operations. in *Digital Image Processing, An algorithmic introduction using Java* 375-427 (Springer, 2008).
9. Romijn, H.J., Sluiter, A.A., Pool, C.W., Wortel, J. & Buijs, R.M. Evidence from confocal fluorescence microscopy for a dense, reciprocal innervation between AVP-, somatostatin-, VIP/PHI-, GRP- and VIP/PHI/GRP-immunoreactive neurons in the rat suprachiasmatic nucleus. *Eur. J. Neurosci.* **9**, 2613-2623 (1997).
10. Lokshin, M., LeSauter, J. & Silver, R. Selective distribution of retinal input to mouse SCN revealed in analysis of sagittal sections. *J. Biol. Rhythms* **30**, 251-257 (2015).
11. Yamasaki, M., *et al.* TARP γ-2 and γ-8 differentially control AMPAR density across schaffer collateral/commissural synapses in the hippocampal CA1 area. *J. Neurosci.* **36**, 4296-4312 (2016).

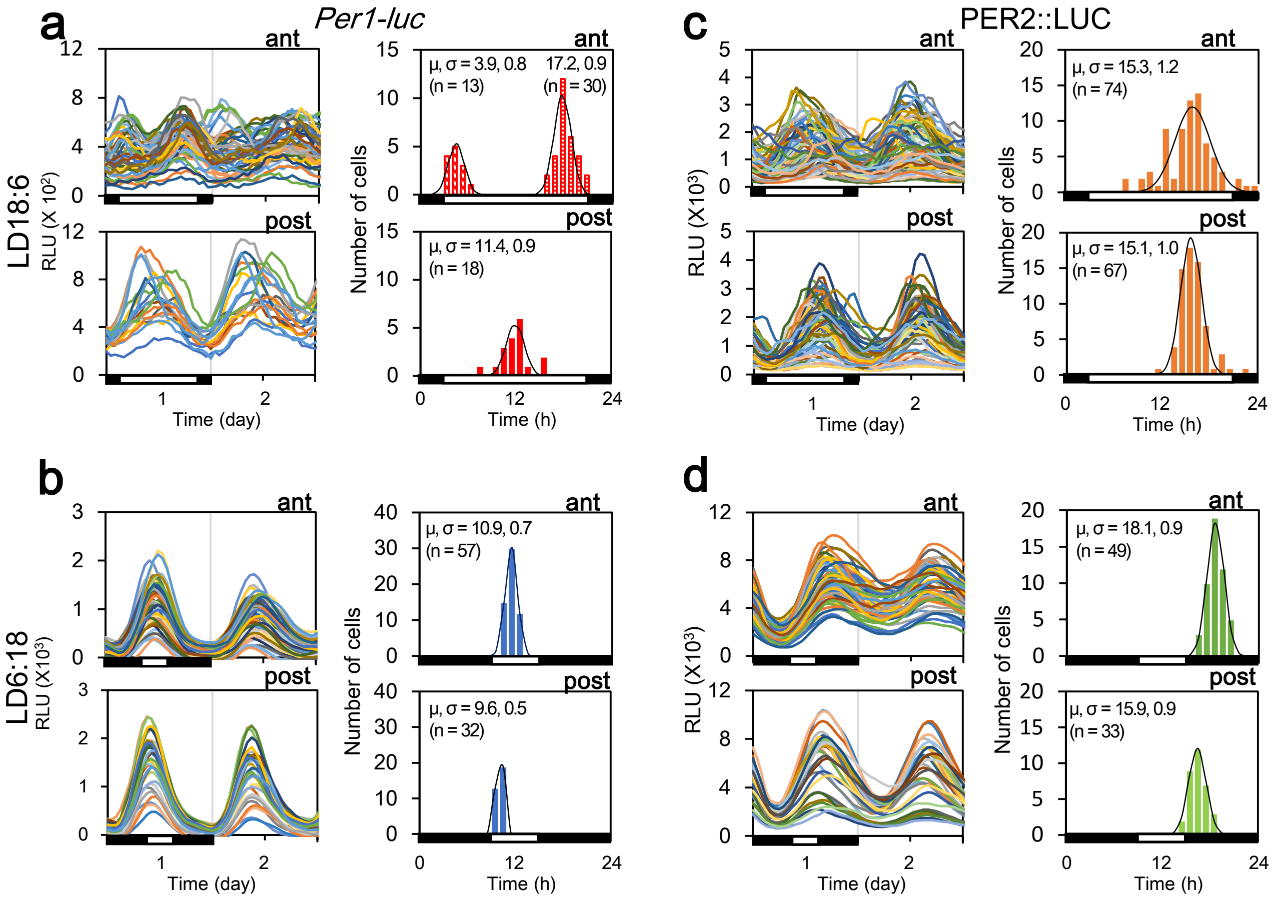

Supplementary Fig. 1. Other examples of circadian phase and variability of circadian peak in the bioluminescence rhythms in cell-size ROI.

a.b.c.d. Forty-eight hour profiles of bioluminescence rhythms in cell-seize ROI and temporal distributions of circadian peaks on the first culture day for *Per1-luc* (a, b) and PER2::LUC (c, d) of the anterior (upper panel) and posterior (lower panel) SCN in each photoperiod. A single or double Gaussian curve fitted to a histogram of circadian phase is indicated by a black curve superimposed on a histogram. Median (μ) and variance (σ) of a Gaussian curve are inserted in each panel.

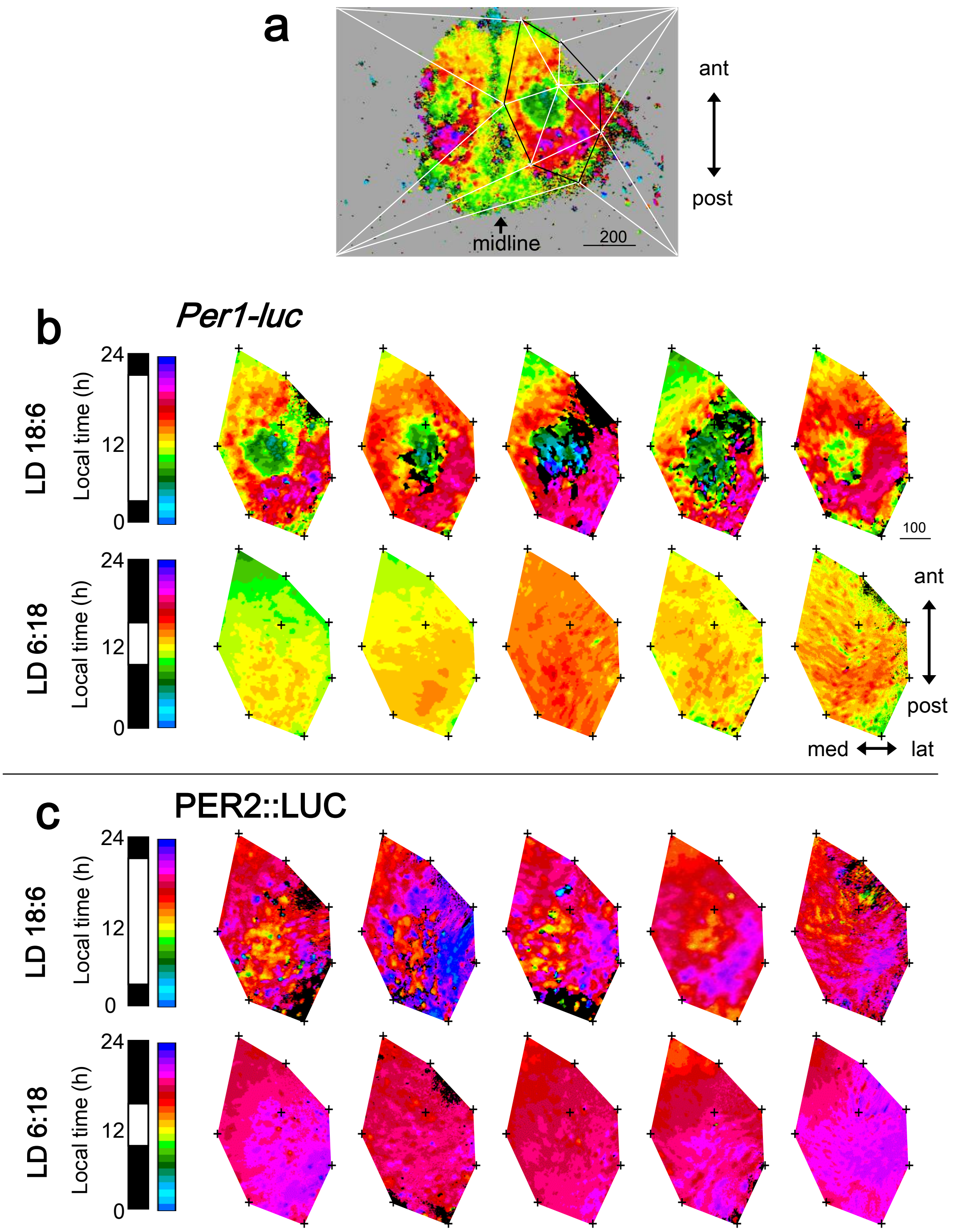

Supplementary Fig. 2. Geometric transformation of an SCN bioluminescence image for statistical analyses on pixel level.

a. An acrophase map of the template SCN for geometric transformation of an SCN bioluminescence image. Black and white lines in the panel indicate the margin of the SCN and Delaunay triangles, respectively. **b, c.** Acrophase maps of five individual transformed SCN slices are illustrated for the *Per1-luc* (b) and PER2::LUC (c) rhythms in LD18:6 (upper) and LD6:18 (lower). Vertical black and white bars indicate the light and dark phases of the photoperiod, and acrophase is shown by pseudocolor**.** Black crosses are the reference points of transformed SCN. Scale bar, 100 µm.

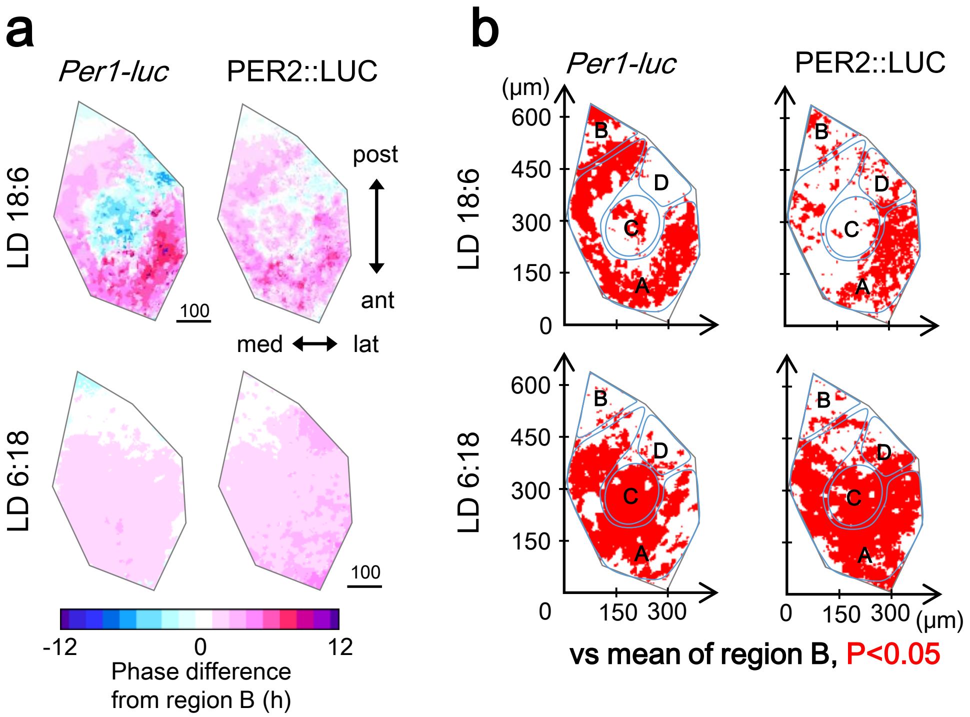

Supplementary Fig. 3. Regional differences in the acrophases of circadian *Per1-luc* and PER2::LUC rhythms in five individual SCN slice.
a. Mean achrophases of circadian *Per1-luc* (left) and PER2::LUC (right) rhythms in LD18:6 (upper) and LD6:18 (lower). Acrophase in each pixel is expressed relative to the mean acrophase in the region B of each slice (n = 5). A pseudo-color bar indicates the phase-difference in hour for phase-advance with a negative sign and for phase-delay with a positive sign. **b.** Statistical significance of the phase differences was demonstrated in an SCN map. Red marks indicate the pixels in which the phase are significantly different from the mean acrophase in the region B (t-test, P<0.05).

Supporting movie 1. *Per1-luc* bioluminescence throughout a horizontal SCN slice of the mouse exposed to LD18:6.

Bioluminescence images of 1-h interval on day 1, 2 and 3 in culture. The bottom and top of the image indicate the anterior and posterior SCN, respectively.

Supporting movie 2. *Per1-luc* bioluminescence throughout a horizontal SCN slice of the mouse exposed to LD6:18.

Bioluminescence images of 1-h interval on day 1, 2 and 3 in culture. The bottom and top of the image indicate the anterior and posterior SCN, respectively.

Supporting movie 3. PER2::LUC bioluminescence throughout a horizontal SCN slice of the mouse exposed to LD18:6.

Bioluminescence images of 1-h interval on day 1, 2 and 3 in culture. The bottom and top of the image indicate the anterior and posterior SCN, respectively.

Supporting movie 4. PER2::LUC bioluminescence throughout a horizontal SCN slice of the mouse exposed to LD6:18.

Bioluminescence images of 1-h interval on day 1, 2 and 3 in culture. The bottom and top of the image indicate the anterior and posterior SCN, respectively.
